# New insights into autophagy in inflammatory subtypes of asthma

**DOI:** 10.3389/fimmu.2023.1156086

**Published:** 2023-04-06

**Authors:** Hongna Dong, Wei Yang, Wei Li, Simin Zhu, Ling Zhu, Peng Gao, Yuqiu Hao

**Affiliations:** ^1^ Department of Respiratory Medicine, The Second Hospital of Jilin University, Changchun, Jilin, China; ^2^ Department of Immunology, College of Basic Medical Sciences, Jilin University, Changchun, China

**Keywords:** asthma, autophagy, biomarker, therapeutic target, inflammation

## Abstract

Asthma is a heterogeneous airway disease characterized by airway inflammation and hyperresponsiveness. Autophagy is a self-degrading process that helps maintain cellular homeostasis. Dysregulation of autophagy is involved in the pathogenesis of many diseases. In the context of asthma, autophagy has been shown to be associated with inflammation, airway remodeling, and responsiveness to drug therapy. In-depth characterization of the role of autophagy in asthma can enhance the understanding of the pathogenesis, and provide a theoretical basis for the development of new biomarkers and targeted therapy for asthma. In this article, we focus on the relationship of autophagy and asthma, and discuss its implications for asthma pathogenesis and treatment.

## 1 Introduction

Asthma is a chronic airway inflammatory disease characterized by airway remodeling, airway hyperresponsiveness (AHR), and increased mucus secretion (1). It is a heterogeneous disease with multiple phenotypes and complex pathogenetic mechanisms. Various inflammatory cells and inflammatory mediators have been implicated in the pathophysiology of asthma. Adaptive and innate immunity play an indispensable role in the pathogenesis of asthma (2, 3). Corticosteroids are still the first-line treatment for asthma. In recent years, significant advances have been made in the identification of high Th2-type asthma-specific biomarkers and the development of new biologics (4, 5). However, treatment of low Th2 asthma and severe asthma infiltration remains a challenge. Asthma is classified as eosinophilic asthma (EA) and neutrophilic asthma (NA), paucigranulocytic asthma and mixed granulocytic asthma based on the proportion of inflammatory cells in the induced sputum. Clinically, EA shows good response to glucocorticoid therapy and newer biologic agents, while there is a paucity of very effective therapeutic agents for NA. This is likely attributable to the different pathogenesis of these asthma subtypes. Therefore, better elucidation of the molecular biology of different asthma inflammatory subtypes is a key research imperative to help identify novel biomarkers and more effective therapeutic targets.

Autophagy is an evolutionarily conserved catabolic process for digesting organelles and cytoplasmic components in lysosomes. Autophagy includes macroautophagy (hereinafter referred to as autophagy), microautophagy, and chaperone-mediated autophagy. Generally speaking, autophagy is macroautophagy, which is a metabolic process of eukaryotic cells and is essential for the maintenance of cellular homeostasis (6). Autophagy involves the following cellular processes: initiation, vesicle nucleation, elongation, membrane elongation, closure, maturation, and degradation. A stable ULK-inducible complex induces autophagosome formation during yeast initiation, and mTORC1 regulates its activity in different nutritional states (6). The nucleation stage mainly involves the formation of the PIK3C3 complex, and BECN1 is involved in the regulation of this process. Subsequently, autophagosomes are formed through the extension of the phagocyte membrane through the Atg12-Atg5-Atg16 complex and the Atg8/LC3 ubiquitination system. Autophagosomes combine with lysosomes to form autophagolysosomes, which are degraded by hydrolases (6). Autophagy contributes to the identification and clearance of infectious pathogens (7), and participates in the occurrence and development of various diseases by regulating inflammation, including cancer, cardiovascular diseases, and neurodegenerative diseases (8). In addition, LC3-associated phagocytosis (LAP) is a novel and unique form of LC3-dependent process of non-canonical autophagy that accelerates the degradation of phagosomes by promoting phagosomal and lysosomal fusion, regulates immunity and inflammation, and clears dead cells (9–11). Selective mitochondrial autophagy refers to mitochondria-specific autophagy that occurs in defective mitochondria following injury or stress (12). Research suggests that mitochondrial autophagy is a factor in asthma airway inflammation and a potential therapeutic target (13, 14). In recent years, several studies have shown the significance of regulation of autophagy in the context of asthma.

## 2 Autophagy and asthma

Aberrant expression of autophagy plays an important role in asthma. *ATG5* single nucleotide polymorphism is closely associated with the development of childhood asthma (15). Autophagy-related genes are important in the immune microenvironment of asthma and may modulate the treatment outcomes. Autophagy-related genes have good classification function, as patients in the low autophagy subtype group were found to have more severe, glucocorticoid-resistant, and poorly controlled asthma (16). In a house dust mite (HDM)-induced asthma model, chloroquine was shown to attenuate airway inflammation, AHR, mucus and collagen production (17). In addition, autophagy also plays a role in severe asthma, as patients with severe asthma were found to have elevated levels of autophagy in peripheral blood and sputum granulocytes compared to those with non-severe asthma and healthy controls (18). Mice with severe asthma were found to have higher levels of autophagy compared to mice with relatively mild ovalbumin (OVA)-induced asthma (19). These findings suggest an important value of autophagy in the development and prognosis of asthma, opening up new directions for asthma treatment.

## 3 Autophagy and immune and non-immune cells in asthma

**Figure 1:**
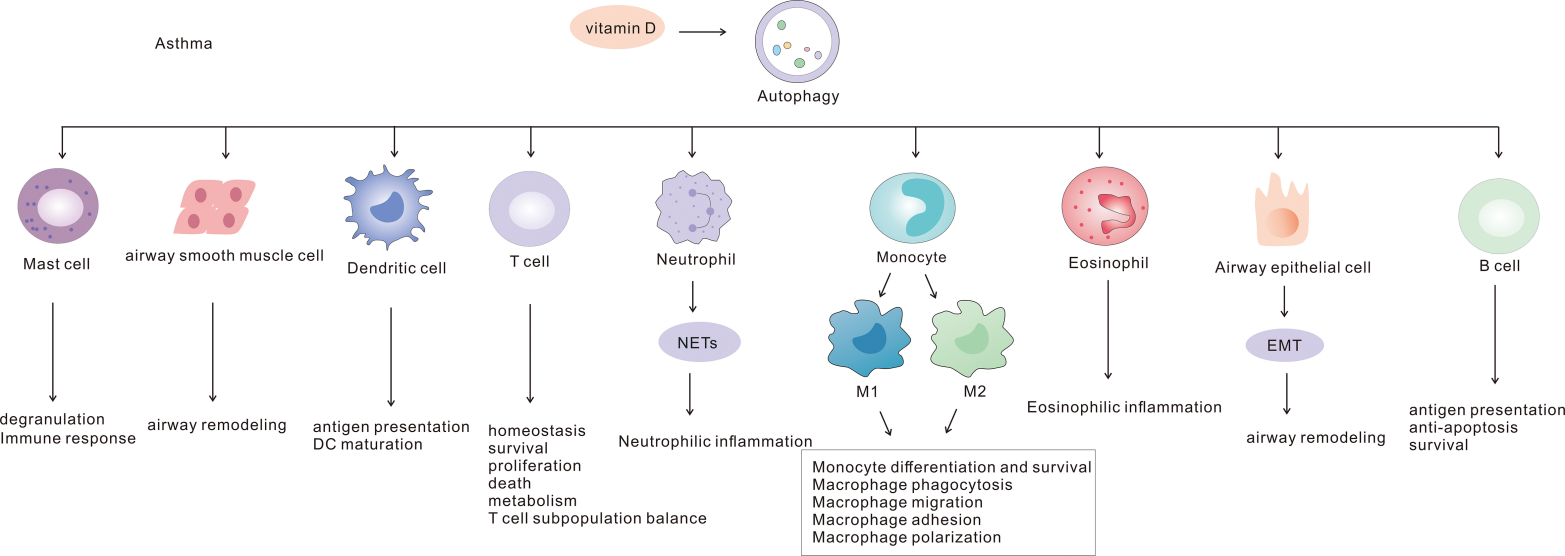
Mechanism of autophagy in asthma.

Autophagy is involved in the pathogenesis of asthma through interactions with a variety of immune and non-immune cells (**Figure 1**). In this paper we summarize the molecular mechanisms of the role of autophagy in asthma and its potential as a therapeutic target.

### 3.1 Autophagy and T cells

Differentiation of various T-cell subsets and the cytokines produced by them play an important role in different asthma inflammatory subtypes. The pathogenesis of EA is dominated by Th2 cells, whereas Th1 and Th17 cells play a dominant role in NA. Autophagy plays a vital role in T cell metabolism, and PIK3C3 deficiency impairs T cell metabolism and inhibits differentiation to Th1. PIK3C3 has a positive effect on aerobic glycolysis and maintenance of mitochondrial respiration in T cells (20). Autophagy is an essential regulatory mediator in T cell homeostasis, survival, proliferation, death, and functional roles (21–27). Autophagy controls the differentiation and activity of CD8+ T cells, and defective autophagy was found to result in a diminished immune response to secondary influenza virus infection (28). In addition autophagy is activated in activated CD4+ T cells and the level of autophagy is higher in Th2 than in Th1 (29). mTOR is a serine/threonine kinase that plays an important role in regulating cell growth, proliferation, aging and longevity (30, 31). mTOR participates in the pathogenesis of various diseases such as liver disease, breast cancer, acute lung injury, Alzheimer’s disease, and asthma by regulating autophagy (32–36). mTOR was shown to be associated with asthma status. Serum mTOR levels were higher in asthmatic patients than in healthy controls during acute exacerbation, but not during remission. In addition, mTOR showed a positive correlation with Th1/Th2, Th17/Tregs imbalance (37). Moreover, the mTOR inhibitor rapamycin was found to increase the proportion of Treg cells in mesenchymal stem cells and decrease the proportion of Th1 cells, while there was no significant change in Th17 cells (38). PI3K/AKT is an upstream pathway of mTOR and is closely related to airway inflammation. IL-27 was shown to attenuate airway remodeling in OVA asthmatic mice through the PI3K/AKT pathway (39). In HDM-stimulated asthmatic mice, brahma-related gene 1 inhibition of PI3K/AKT was found to exacerbate airway inflammation, whereas PI3K inhibitor attenuated this effect (40). In mice with OVA-induced allergic airway inflammation, TLR2 was found to induce autophagy through PI3K/AKT to promote airway inflammation. Moreover, the autophagy inhibitor 3 methyladenine alleviated inflammation (41). Luteolin and pingchuanning decoction improves asthma inflammation by inhibiting autophagy *via* activation of PI3K/AKT/mTOR pathway (42, 43). In addition, LAP supports the presentation of major histocompatibility complex (MHC)-II class antigen to helper CD4+ T cells which are key cells in the pathogenesis of asthma (44–46). In addition, LAP dysregulation can lead to autoimmune disease and inflammation (10). LAP was shown to stimulate macrophage dysregulation in chronic obstructive pulmonary disease (COPD) and cigarette smoke extracts, which may be involved in the pathogenesis of COPD (47). However, there is a need for further studies on its role in the pathogenesis of asthma.

Type 2 innate lymphoid cells (ILC2) are involved in the pathogenesis of Th2 asthma, and autophagy is involved in ILC2 activation and immune homeostasis. Lack of autophagy in ILC2 promotes glycolysis, inhibits fatty acid oxidation and TCA cycle, suppresses Th2 cytokine production, and attenuates asthma AHR (48). Sepsis caused by Methicillin-resistant Staphylococcus aureus (MRSA) infection was associated with elevated levels of autophagic protein and elevated serum levels of interferon-gamma (IFN-γ) and interleukin (IL)-17; in addition, rapamycin enhanced autophagy to reduce the ratio of Th1 and Th17 cells and promote survival in septic mice (49). The complex interplay between autophagy and T cell subsets and cytokines has given rise to an in-depth consideration of their role in asthma.

### 3.2 Autophagy and monocytes/macrophages

Macrophages are the main immune cells in asthma and are involved in phagocytosis, secretion of inflammatory factors, antigen presentation, and regulation of the immune response (50, 51). Studies have shown impaired phagocytosis of macrophages in children with non-eosinophilic asthma and poorly controlled asthma (52, 53). Macrophages can be classified into two phenotypes, i.e., classically activated M1 and substitution-activated M2, and these two macrophage states reflect Th1-Th2 polarization. M1 is stimulated by IFN-γ cytokines to produce Th1 cytokines (IL-6, IL-1β) that promote neutrophil inflammation, while M2 is stimulated by IL-4 and IL-13 to produce eosinophil-promoting cytokines (IL-10, TGF-β) (54). This has also been verified in animal models of asthma, where M1 polarisation was predominant in farm dust extracts-induced non-allergic asthma and where Th1 and Th17 cells and neutrophil infiltration were predominant. In contrast, in house dust mite-induced allergic asthma, M2 polarisation was predominant and Th2 cells and eosinophils were increased (55).

Paucigranulocytic asthma is a milder form of asthma characterized by macrophages as the predominant cell type. The molecular mechanisms underlying this type are not well understood. Macrophage polarization induces Th1 and Th2 cytokines involved in the pathogenetic mechanism of EA and NA. In contrast, autophagy has regulatory effects on the differentiation and survival of monocytes (56), phagocytosis, migration and adhesion functions of macrophages, macrophage polarization and bacterial killing. High glucose conditions can inhibit autophagy and promote macrophage migration and adhesion, providing a new strategy for the treatment of diabetic nephropathy (57). Autophagy is impaired in obese mice, and inhibition of macrophage autophagy promotes their polarization to M1 phenotype (58). In addition, lactobacillus induces autophagy by modulating Th1/Th2 cytokine balance to enhance mononuclear phagocyte response to Mycobacterium tuberculosis infection (59). Inhibition of autophagy enhances phagocytosis of MRSA in MRSA-infected mice (60). *Pseudomonas aeruginosa* infection promotes macrophage autophagy and limits macrophage intracellular killing, probably by activating NLRP3 vesicles and reducing the production of reactive oxygen species (ROS) and nitric oxide (NO); thus, inhibition of autophagy can promote clearance of bacteria (61, 62). In contrast, in Mycobacterium tuberculosis infection, autophagy inhibits the survival of Mycobacterium tuberculosis in a PI3K-dependent manner and contributes to an enhanced immune response (63). The different roles of autophagy may depend on the microenvironment and therefore specific studies are needed for different diseases.

### 3.3 Autophagy and dendritic cells

Dendritic cells play an important role in adaptive immunity because of their antigen-presenting role. Autophagy has multiple effects on the functional regulation of DCs. Ho et al. demonstrated that autophagy regulates DC antigen presentation (64). Autophagy promotes the survival of lipopolysaccharide-induced DCs under hypoxic conditions (65), and defective autophagy protein VPS34 increases MHC-I and class II antigen presentation by DC cells (66). In contrast, in a DC study of ATG5 and ATG7 deficiency, autophagy was shown to inhibit MHC-I and promote MHC-II class antigen presentation (67). DC deficiency of ATG5 promotes antiviral immune responses involving CD8+ cells (68). Autophagy in RSV-infected DCs was shown to facilitate CD4 cytokine production and DC maturation, promoting antiviral responses (69, 70).

### 3.4 Autophagy and B cells

The necessity of autophagy for B-cell polarization has yet to be investigated. ATG5 is involved in antigen presentation and B-cell polarization (71). Basal level autophagy is required for B-cell survival (72, 73). Dendritic cells and B-cell autophagy deficiency in toll-like receptor (TLR) 7-deficient mice was shown to promote activation of inflammatory vesicles and organ damage (74). While TLR7 agonists are beneficial in asthma, TLR7 agonists are effective airway diaphoresis substances (75, 76). In OVA-induced asthmatic mice, TLR7 was shown to attenuate airway inflammation by mediating the Nrf2 pathway (77). IL-4 induces enhanced B-cell autophagy in asthmatic mice *via* IAK, which contributes to antigen presentation and anti-apoptosis and is involved in asthma pathogenesis (78). B cells are important in the adaptive immune response and autophagy and its role in asthma deserves to be explored.

### 3.5 Autophagy and mast cells

Mast cells are key effector cells involved in the inflammation, airway hyperresponsiveness, and response to steroid treatment in asthma (79–81). Asthmatics have increased mast cells in airways (82). Mast cell activation correlates with asthma phenotype, and IL-33 and IgE stimulated activation of mast cells correlates with severe neutrophilic and eosinophilic asthma phenotypes, respectively (83). Mast cells were also shown to be associated with the response to corticosteroid treatment; in a study, the inhaled corticosteroid group showed reduction in mast cells in the epithelium and smooth muscle compared to the non-inhaled corticosteroid group (84). Autophagy is involved in mast cell degranulation and ATG7-deficient MC cells show severely impaired degranulation (85, 86). IL-33 expression is elevated in nasal epithelial cells in allergic rhinitis, inhibits autophagy and regulates degranulation and inflammatory factor release in mast cells *via* IL-33/ST2 (87). Mast cell autophagy contributes to defence against pathogenic bacteria. *Pseudomonas aeruginosa* infection promotes autophagy of airway epithelial cells and mast cells and facilitates bacterial clearance (88). Orosomucoid-like-3 (*ORMDL3*) is an asthma susceptibility gene. Studies have shown that ORMDL3 inhibits mast cell activation and degranulation and production of cytokines and chemokines through the activating transcription factor 6-related autophagic pathway, thereby suppressing the immune response (89). Mast cell autophagy is a potential therapeutic target in asthma worthy of future research.

### 3.6 Autophagy and eosinophils

EA is a classic allergic asthma that shows good response to glucocorticoids. The novel immunologic agents (e.g., anti-IL-4 monoclonal antibody, anti-IL-5 monoclonal antibody, anti-IgE monoclonal antibody) are also useful in this type of asthma. ATG5 is the main protein of the autophagic process. *In vivo* and *in vitro* experimental studies have shown that ATG5 deficiency leads to inhibition of eosinophil proliferation and differentiation, and enhancement of eosinophil extracellular traps formation and degranulation, promoting bacterial clearance (90). In addition, autophagy inhibits the release of the content of mature eosinophils, which may help reduce inflammation in EA (91). Impaired autophagy in obese HDM-sensitized asthmatic mice was found to aggravate airway inflammation and increase eosinophilic airway inflammation in ATG5-deficient mice with resistance to dexamethasone (92). Paradoxically, autophagy protein expression is increased in the peripheral blood of patients with severe asthma and IL-5 induces autophagy to promote the production of eosinophil cationic protein and eosinophil airway inflammation (18). Studies in asthmatic animals show that autophagy is associated with a severe eosinophil phenotype and that anti-IL-5 has a beneficial effect on asthma treatment by reducing autophagy (19). Moreover, the mTOR inhibitor rapamycin was shown to inhibit eosinophil differentiation in OVA-induced allergic asthma mice (93). The complex relationship between autophagy and eosinophils is inconclusive and may be related to the different pathogenesis of different asthma phenotypes, which remains to be elucidated.

### 3.7 Autophagy and neutrophils

Neutrophilic asthma is typically difficult to treat owing to resistance to glucocorticoid therapy and lack of other effective drugs. Moreover, biologic agents are mainly effective in EA. Autophagy has a multifaceted role in regulating neutrophil function. A study showed that genetic polymorphisms in the autophagy genes *ATG5* and *ATG7* are associated with NA inflammation (94). Autophagy is essential for neutrophil maturation and function. Defective autophagy leads to accumulation of immature neutrophils and impaired function, and autophagy can facilitate the differentiation process by inhibiting glycolysis and promoting energy metabolic shifts during lipolysis (95). In contrast, in immature neutrophils, autophagy has an inhibitory effect on neutrophil differentiation, possibly due to regulation of the p38-mTORC1 signaling pathway. The regulation of neutrophils by autophagy may be due to the regulation of multiple molecular mechanisms, and further experimental studies are needed (96). In addition, autophagy promotes neutrophil degranulation and is an important mediator in neutrophil inflammation (97). ATG5-deficient mice exacerbate neutrophil airway inflammation and cause exacerbation of steroid-resistant asthma involving IL-17A (98). Rapamycin can inhibit IL-17A production and suppress neutrophil inflammation (99).

Neutrophil extracellular traps (NETs) are trap networks consisting of DNA and various enzymatic proteins that form after neutrophils are stimulated to form cytoplasmic extracellular traps. It has an important anti-infective function and has a crucial role in asthma. The relationship between autophagy and NET is still controversial. Autophagy has been shown to be necessary for NET formation and to promote the formation of NETs (100). In an autophagy gene *ATG5*-deficient mouse model of senescence, TLR2 ligands were found to reduce NETs formation in neutrophils (101). Moreover, NETs formation was shown to be associated with autophagy and autophagy inducers promoted spontaneous release of NETs, further confirming that this relationship also exists in the OVA-induced asthma model (102). Increased levels of peripheral blood neutrophil autophagy protein were observed in patients with severe asthma relative to those in patients with non-severe asthma; in addition, a positive correlation between NET and autophagy was observed which may be involved in the pathogenesis of severe asthma by enhancing the eosinophil inflammatory response and epithelial cell damage (103). NET formation induced by phorbol myristate acetate (PMA) requires the involvement of autophagy and superoxide (100). Studies have also suggested a key role of autophagy in anti-neutrophil cytoplasmic antibody (ANCA)-associated vasculitis, and autophagy was found to promote anti-LAMP-2 antibody and ANCA antibody-induced NET production (104, 105). However, PMA-stimulated neutrophil studies in ATG5-deficient mice showed that NET formation does not require the involvement of autophagy (106). Interestingly, it has been shown that autophagy has no significant effect on NETs generation. Autophagy gene *ATG5* is not necessary for extracellular traps of neutrophils and eosinophils (97). Autophagy and NETs are associated with airway inflammation in severe asthma, and further studies are required for in-depth characterization of the relationship of autophagy and NETs with asthma. Mice lacking autophagy showed increased inflammation in IL-17-mediated bands of lung neutrophils. Absence of autophagy in lung CD11c cells induces neutrophil airway inflammation and airway hyperresponsiveness leading to exacerbation of asthma (98). Targeting autophagy-dependent NETs is a novel therapeutic strategy for asthma.

### 3.8 Autophagy and airway epithelial cells

Autophagy plays an important role in airway epithelial regeneration, lung development and morphogenesis (107, 108). IL-13 expression is elevated in EA, which can activate airway epithelial cell autophagy to promote mucus secretion and production of ROS (109). In addition, experiments in OVA-induced asthma mouse models and airway epithelial cells demonstrated that TGF-β3 promotes MUC5AC production for mucus secretion by regulating autophagy (110). Interestingly, specific knockout of mTOR in the airway epithelium induces autophagy and promotes allergic airway inflammation in mice (36). Epithelial mesenchymal transition (EMT) plays a key role in promoting asthma airway remodeling. In OVA-induced asthmatic mice, azithromycin was found to inhibit EMT and improve asthma airway remodeling (111). The link between autophagy and EMT has been widely studied in the context of cancer treatment and is gaining attention in asthma (112, 113). Autophagy is a key link in mesenchymal-to-epithelial transition (114). *In vivo* and *in vitro* studies have shown that FSTL1 activates autophagy and promotes EMT, thereby facilitating asthma airway remodeling (115). Further studies are required to demonstrate the mechanisms linking autophagy and EMT in asthma and to provide new strategies for asthma treatment.

### 3.9 Autophagy and airway smooth muscle cells

**Figure 2:**
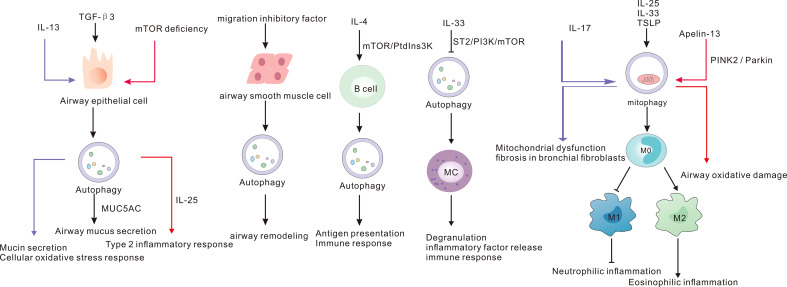
The cells and cytokines involved in asthma and autophagy.

Airway smooth muscle is an important cell for airway remodeling in asthma. Azithromycin is one of the promising drugs for the treatment of NA; however, its exact mechanism of action remains to be elucidated. In addition, azithromycin was shown to reduce the proliferation of airway smooth muscle cells and induce autophagy (116). Knockdown of *p62* in chronic asthma model inhibited the proliferation and migration of airway smooth muscle cells (117). Autophagy has important implications for the pathogenesis of cells and cytokines involved in asthma (**Figure 2**). Further studies are required for in-depth elucidation of its role.

### 3.10 Autophagy and vitamin D

Several studies have suggested that vitamin D may be an important mediator in different asthma phenotypes. However, there is no clear consensus regarding its specific mechanism of action. In a clinical trial including 54 adults with asthma, elevated serum vitamin D levels were associated with reduced AHR, improved lung function and increased sensitivity to glucocorticoids and exercise-induced bronchoconstriction (118, 119). In addition, vitamin D may be involved in the pathogenesis of asthma associated with obesity, and Vitamin D deficiency was shown to impair lung function in these patients (120). Vitamin D deficiency was also implicated as a factor in worsening of asthma control (121, 122). In asthmatic mice, administration of vitamin D was found to inhibit AHR and suppress inflammation (123). Moreover, a study showed that vitamin D and vitamin D receptor can regulate autophagy (124), although the mechanism of action in the context of asthma remains to be elucidated. Infection is a common trigger for worsening of asthma, and vitamin D plays a critical role in lung infection. In *Aspergillus fumigatus*-infected mice, the survival rate of vitamin D-sufficient mice was higher than that of the deficient group (125, 126). Vitamin D may protect against damage caused by *Aspergillus fumigatus* infection by reducing the expression of inflammatory factors such as NF-kB and IL-1β, IL-6 and inhibiting lysosome formation to reduce autophagy (125, 126). Autophagy-related proteins ATG5 and Becilin1 were increased in the OVA-induced asthma mouse model in the vitamin D-deficient group, compared with the vitamin D-sufficient group (127). In respiratory syncytial virus-induced asthmatic mice, vitamin D has the same effect of reducing inflammation, and it may act through the Notch1-HIF-1α signaling pathway to promote autophagy (128). Vitamin D has an important role in asthma, and the link between it and autophagy is worth exploring to provide new ideas for targeted asthma therapy.

## 4 miRNA mediated autophagy in asthma

MicroRNAs (miRNAs) represent a new tool for targeted therapy for asthma. The link between miRNAs and autophagy in asthma has received increasing attention. Studies have demonstrated the involvement of miRNA-targeted autophagy in the pathogenesis of asthma. MiR-192-5p, miR-20a-5p, miR-34/miR-449, miRNA-335-5p, and miR-30a inhibit autophagy in asthma through different pathways. Overexpression of miR-192-5p was found to reduce the expression of MMP-16 and ATG7, attenuate airway remodeling and inhibit autophagy in asthmatic mice (129). Overexpressed miR-20a-5p acts as an autophagy inhibitor, targeting ATG7 to inhibit autophagy, apoptosis, fibrosis and airway inflammation in asthma (130). MiR-335-5p and miR-30a target ATG5 to suppress asthma inflammation (131, 132). MiR-34/miR-449 overexpression promotes Nur77 nuclear translocation and inhibits autophagy by downregulating IGFBP-3, reducing airway fibrosis and inflammation (133). Studies of the relation between miRNA and autophagy provide a basis for the elucidation of the mechanism of asthma. However, further studies are required to unravel the detailed process of miRNA regulation of autophagy to provide new ideas for the development of targeted drugs for asthma treatment.

## 5 Autophagy and asthma treatment

Corticosteroids are the drugs of choice for asthma treatment; however, they are not always effective, and some patients develop steroid resistance. Respiratory tract infections are a common cause of acute exacerbations of asthma. In recent years, several drugs targeting autophagy (inhibiting autophagy or activating autophagy) have been identified for their potential use in asthma (**Table 1**). However, there are some contradictory results, and there may be many mechanisms involved, which still needs further research. *In vitro*, budesonide and simvastatin inhibited mTOR-mediated autophagy in macrophages, decreased Beclin-1 and LC3 expression, increased p62 and IL-10 expression, inhibited autophagy and reduced asthma inflammation (135). In contrast, budesonide and simvastatin were found to activate autophagy through other pathways to reduce asthma inflammation. Budesonide can activate autophagy to exert anti-rhinovirus effect and has a protective effect against asthma attacks (138). Simvastatin activates autophagy in bronchial smooth muscle cells (BSMCs) to inhibit airway inflammation and airway remodeling for the treatment of asthma (137). In addition, a novel biologic anti-IL-5 antibody significantly reduced LC3 II expression in lung homogenates (19). High-dose luteolin, yeast-fermented prebiotic, ketamine and α1-antitrypsin inhibit autophagy in lung tissue ameliorating asthmatic mice (42, 134, 136, 144). Chinese herbal medicines can also regulate autophagy and can be potentially used for asthma treatment. Paeoniflorin modulates mitochondrial function by suppressing autophagy and reducing the expression of pro-inflammatory factors to alleviate asthmatic inflammation (145). Astragalin can alleviate ROS-promoted bronchial fibrosis by inhibiting the formation of airway autophagosomes (142). Acupuncture was shown to reduce airway inflammation and AHR by modulating endoplasmic reticulum stress and CD4+ T lymphocyte differentiation by inhibiting ATG5-mediated autophagy (146).

**Table 1:** Mechanisms of action of drug-mediated autophagy on allergic airway inflammation *in vivo* and *in vitro*.

Drug	Species	tissue	Cell line	Autophagy-related target	Signal pathway	Effect on autophagy	Function	Ref
α1-Antitrypsin	mouse	–	BEAS-2B	LC3BII/LC3BI, BECN1, SQSTM/p62	–	Inhibition	α1-Antitrypsin attenuates asthmatic inflammation and oxidative stress by inhibiting autophagy.	(134)
budesonide and simvastatin	–	–	PBMC	beclin-1, LC3, p62	–	Inhibition	Glucocorticoids and statins reduce asthma inflammation by promoting IL-10 production by inhibiting autophagy in macrophages.	(135)
Luteolin	mouse	lung	–	LC3B, p62,PI3K,AKT	PI3K/Akt/mTOR, Beclin-1-PI3KC3	Inhibition	Inhibition of autophagy in lung tissue of asthmatic mice by luteolin ameliorates airway inflammation and reduces airway mucus secretion and collagen deposition in allergic asthmatic mice.	(42)
Yeast Fermentate Prebiotic	mouse	lung		ATG5, Beclin1, LC3BII/I	–	Inhibition	YFP exerts anti-inflammatory and antiasthma effects by inhibiting autophagy and regulating oxidative stress in asthmatic mice.	(136)
Simvastatin	mouse	lung	BSMCs	ATG5, LC3B, Beclin1	–	Activation	Simvastatin increases autophagy protein expression in the lung tissue of asthmatic mice, and autophagy inhibits inflammation and airway remodeling in activated BSMCs *in vitro*.	(137)
Budesonide	mouse	lung	HeLa cells, HeLa cells	P62, LC3	–	Activation	Budesonide can increase mitochondrial reactive oxygen species levels, activate autophagy, inhibit inflammatory response and IL-1β production, and participate in anti-rhinovirus and anti-inflammatory effects.	(138)
Cycloastragenol	mouse	lung	–	LC3B, p62, Beclin 1	–	Inhibition	Cycloastragenol inhibits autophagy levels and exerts anti-inflammatory effects in asthmatic mice.	(139)
Qingfei oral liquid	mouse	lung	16 HBE cells	LC3B, Beclin-1, p62, Atg5	mTOR	Inhibition	QF may alleviate inflammation induced by respiratory syncytial virus infection in asthmatic mice by inhibiting autophagy through mTOR pathway.	(140)
Wuhu Decoction	mouse	lung	DC	LC3-II, Beclin-1, LC3-I	AMPK/ULK1	Activation	Wuhu Decoction promotes autophagy in lung tissue DCs through AMPK/ULK1 signaling pathway to alleviate RSV-induced asthma inflammation.	(141)
Pingchuanning decoction	rat	lung	RTE	LC3‐I, LC3‐II, P62, beclin‐1, Atg3, Atg5, Atg7	PI3K/Akt/mTOR, HMGB1/TLR4/NF-κB	Inhibition	Pingchuanning decoction attenuates airway inflammation by inhibiting autophagy through PI3K/Akt/mTOR signaling pathway in asthma.	(43)
Astragalin	mouse	lung	BEAS-2B	beclin-1, LC3A/B	–	Inhibition	Astragalin inhibits the formation of autophagosomes in the airway epithelium and alleviates oxidative stress-induced airway fibrosis.	(142)
Trehalose	–	–	Normal human tracheobronchial epithelial	LC3 I, LC3 II, ATG5	–	Activation	Trehalose-induced autophagy impairment reduces IFN-λ1 expression, impairs antiviral responses, and increases the risk of HRV-infection in normal human primary airway epithelial cells.	(143)
Ketamine	Mouse	lung	–	LC-I, LC-II, Beclin-1	mTOR	Inhibition	Ketamine attenuates allergic airway inflammation by inhibiting autophagy through the mTOR signaling pathway.	(144)

## 6 Prospects and conclusion

Collectively, the available evidence suggests a key role of autophagy in the pathogenesis of asthma. Autophagy abnormality leads to the process of asthmatic airway inflammation and airway remodeling, indicating its promising role as a therapeutic target. This review also discusses the complex relationship between miRNAs and autophagy in asthma pathogenesis. However, there is a paucity of research on the underlying mechanism by which miRNA regulates autophagy in asthma. Most of the available evidence emanates from *in vitro* and *in vivo* preclinical studies. Development of targeted therapy for asthma is a key research imperative. The process of autophagy may be involved in regulating the immune response in different asthmatic inflammatory subtypes by influencing the antigen presentation, and secretion of cytokines by cells involved in asthma pathogenesis (e.g., B cells, T cells, macrophages, mast cells, neutrophils, and eosinophils). Based on the central role of autophagy in innate and adaptive immune responses, autophagy may play a beneficial or detrimental role in asthma, depending on the cellular microenvironment in asthmatic inflammatory subtypes. Further studies are required to unravel the specific mechanisms of the regulatory role of autophagy in asthma.

## Author contributions

YH and PG designed and conceived the study, and reviewed the manuscript; WY and HD wrote and revised the manuscript; WL, SZ, and LZ collected the data and created the figures and tables. All authors read and approved the final manuscript for publication.
